# P-328. PrEP Utilization Patterns and Indications in a Cohort of HIV-Negative Individuals

**DOI:** 10.1093/ofid/ofaf695.547

**Published:** 2026-01-11

**Authors:** Richard A Elion, Kenneth H Mayer, Rachel Scott, Aniruddha Hazra, Helen Koenig, Isobel McEwen, Chris Nguyen, Kristin Baker, Olayemi Oladapo, Yenyen Tran, Joshua Gruber, Brett Shannon, Janna Radtchenko

**Affiliations:** Trio Health, Louisville, Colorado; Harvard Medical School/Fenway Research Institute, Boston, MA; MedStar Health Research Institute, Georgetown University, Washington, District of Columbia; University of Chicago, Chicago, IL; Hospital of the University of Pennsylvania, Philadelphia, PA; Trio Health, Louisville, Colorado; Gilead Sciences, Inc., Foster City, California; Gilead Sciences, Inc, Foster City, California; Gilead Sciences, Inc, Foster City, California; Gilead Sciences, Inc, Foster City, California; Gilead Sciences, Forest City, California; Gilead Sciences, Inc, Foster City, California; Trio Health, Louisville, Colorado

## Abstract

**Background:**

Pre-exposure prophylaxis (PrEP) is effective in preventing HIV, yet many individuals who could benefit remain unprotected. This study assessed the PrEP cascade in a database enriched for populations disproportionately affected by HIV.

**Figure 1:**
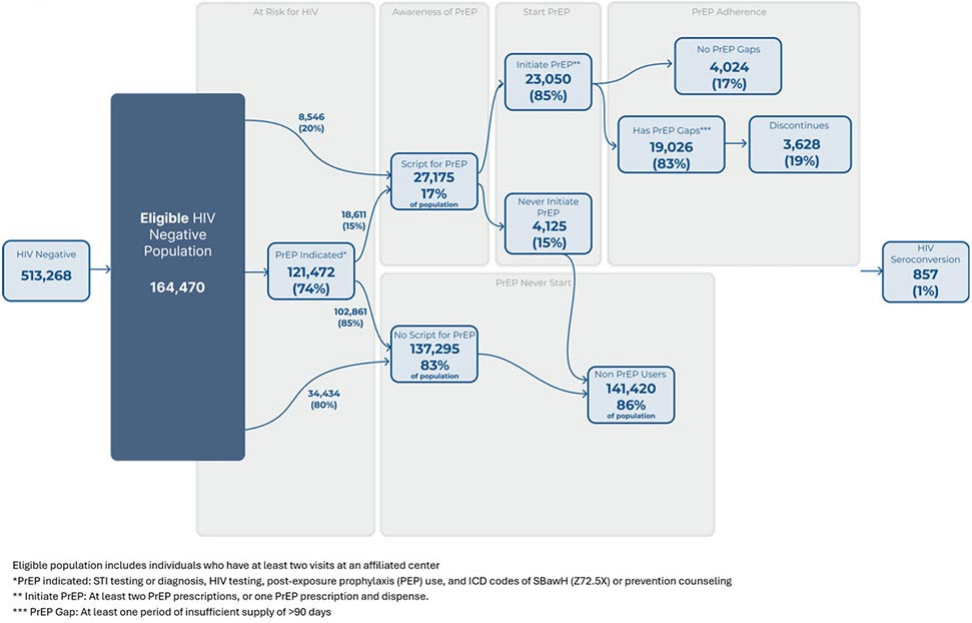
PrEP Cascade Summary

**Table 1:**
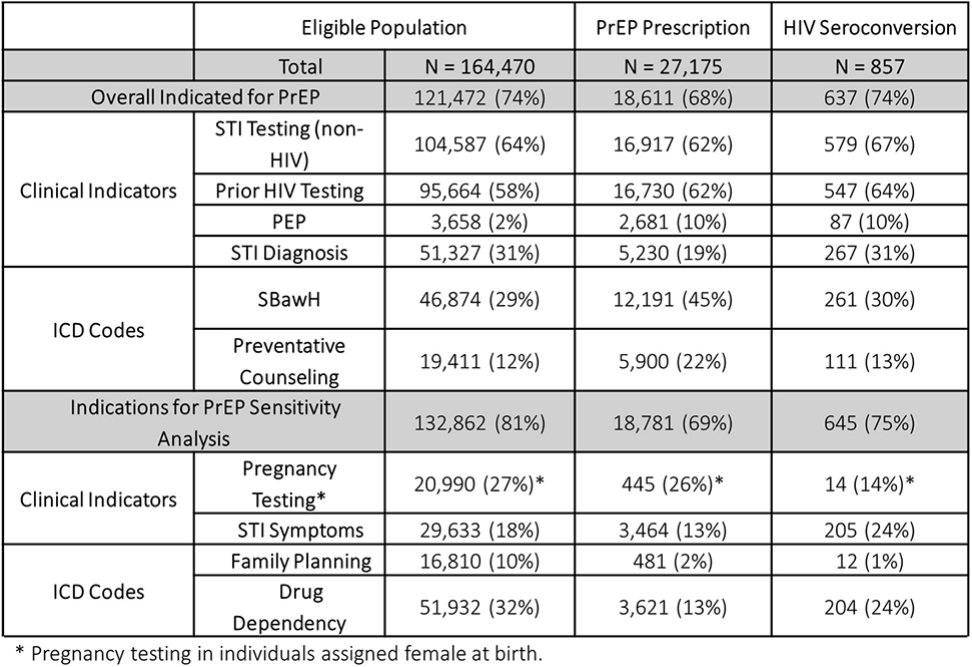
HIV Exposure Classification by PrEP Prescriptions and HIV Seroconversions

**Methods:**

People without HIV (PWoH) with ≥2 visits were evaluated using Trio Health Database from 2016 onward. “PrEP indicated” population was identified based on STI testing or diagnosis, post-exposure prophylaxis (PEP) use, and ICD codes indicating sexual behaviors associated with HIV acquisition (SBawH) or prevention counseling. Gaps (no drug supply >90 days) and discontinuation were analyzed based on prescription and dispensing of emtricitabine/tenofovir or cabotegravir for PrEP (*Figure 1)*. Initiated PrEP was ≥2 prescriptions or ≥1 dispense.

**Table 2:**
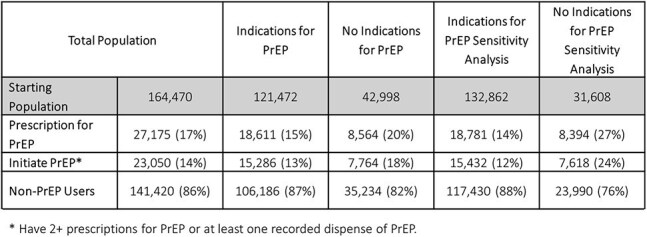
PrEP Utilization by Indicators of PrEP Eligibility

**Results:**

Of 164,470, 17% received a PrEP prescription, of them 85% initiated PrEP. Of PrEP initiators 19% discontinued (*Figure 1*). “PrEP indicated” was 74% of the cohort: 64% had STI testing, 58% HIV testing, 31% STI diagnoses, 2% prior PEP, 29% ICD codes of SBawH, and 12% prevention counseling (*Table 1*). Of ”PrEP indicated”, 15% received PrEP, while individuals without these indicators had higher prescription rates (20%) (*Table 2)*. Sensitivity analysis expanded the criteria, increasing the “PrEP indicated” population to 81% with additional criteria: pregnancy testing (27%), STI symptoms (18%), drug dependency (32%), and family planning (10%) (*Table 1*). One percent (857) of PWoH seroconverted. Individuals prescribed PrEP were more likely to have ICD codes for SBawH (45%) and prevention counseling (22%), while STI diagnoses (31%) and HIV testing (58%) were more common among those who seroconverted, 83% of individuals with PrEP prescriptions had a gap in PrEP.

**Conclusion:**

While many individuals had indicators suggesting benefit from PrEP, a smaller proportion of PWoH received PrEP prescriptions compared to those without such indicators, highlighting possible missed opportunities. Reliance on clinical codes has limitations, as incomplete or inconsistent documentation may lead to incorrect assumptions regarding behavior. Identification of robust clinical and behavioral factors could facilitate effective PrEP intervention, strengthening prevention efforts.

**Disclosures:**

Richard A. Elion, MD, Gilead Sciences: Advisor/Consultant|Gilead Sciences: Grant/Research Support|Trio Health: Employee|ViiV Healthcare: Advisor/Consultant|ViiV Healthcare: Grant/Research Support Kenneth H. Mayer, MD, MPH, Gilead Sciences: Advisor/Consultant|Gilead Sciences: Grant/Research Support|Merck, Inc: Advisor/Consultant|Merck, Inc: Grant/Research Support|Moderna: Grant/Research Support|ViiV Healthcare: Advisor/Consultant|ViiV Healthcare: Grant/Research Support Rachel Scott, MD, MPH, Gilead Sciences, Inc: Advisor/Consultant|Viiv: Advisor/Consultant|Viiv: Grant/Research Support Aniruddha Hazra, MD, Gilead Sciences: Advisor/Consultant|Gilead Sciences: Grant/Research Support|ViiV Healthcare: Advisor/Consultant Isobel McEwen, MS, Trio Health: Analyst Chris Nguyen, PharmD, Gilead Sciences, Inc.: Employee and shareholder Kristin Baker, PA-C, Gilead Sciences, Inc: Ownership Interest Olayemi Oladapo, PharmD, Gilead Sciences, Inc: Ownership Interest|Gilead Sciences, Inc: Stocks/Bonds (Public Company) Joshua Gruber, PhD MPH, Gilead Sciences: Employee and shareholder Brett Shannon, PhD, Gilead Sciences, Inc: Ownership Interest Janna Radtchenko, MBA, Trio Health: Employee

